# FF-HPINet: A Flipped Feature and Hierarchical Position Information Extraction Network for Lane Detection

**DOI:** 10.3390/s24113502

**Published:** 2024-05-29

**Authors:** Xiaofeng Zhou, Peng Zhang

**Affiliations:** School of Electronics and Communication Engineering, Shenzhen Campus of Sun Yat-sen University, Shenzhen 518107, China; zhouxf28@mail2.sysu.edu.cn

**Keywords:** lane detection, deep learning, Flipped Feature Extraction, Hierarchical Position Information Extraction, Deformable Context Extraction

## Abstract

Effective lane detection technology plays an important role in the current autonomous driving system. Although deep learning models, with their intricate network designs, have proven highly capable of detecting lanes, there persist key areas requiring attention. Firstly, the symmetry inherent in visuals captured by forward-facing automotive cameras is an underexploited resource. Secondly, the vast potential of position information remains untapped, which can undermine detection precision. In response to these challenges, we propose FF-HPINet, a novel approach for lane detection. We introduce the Flipped Feature Extraction module, which models pixel pairwise relationships between the flipped feature and the original feature. This module allows us to capture symmetrical features and obtain high-level semantic feature maps from different receptive fields. Additionally, we design the Hierarchical Position Information Extraction module to meticulously mine the position information of the lanes, vastly improving target identification accuracy. Furthermore, the Deformable Context Extraction module is proposed to distill vital foreground elements and contextual nuances from the surrounding environment, yielding focused and contextually apt feature representations. Our approach achieves excellent performance with the F1 score of 97.00% on the TuSimple dataset and 76.84% on the CULane dataset.

## 1. Introduction

Thanks to the continuous advancements in deep learning and computer vision, neural network-based lane line detection has significantly improved and achieved excellent performance. Lane detection plays a crucial role in intelligent automated driving, enabling vehicles to accurately perceive their surroundings and facilitate navigation planning. The essence of successful lane detection lies not only in identifying the presence of lane lines but also in precisely determining their spatial locations on the road. As the demands and expectations for autonomous driving increase, more challenges arise for achieving high accuracy and generalization capability in lane line detection. It is essential to develop methods and techniques that can handle various scenarios and environmental conditions effectively. These challenges emphasize the importance of further improving the accuracy and robustness of lane line detection systems.

Compared to traditional methods like Hough Line [1,2], deep learning-based methods have shown significant improvements in detection effectiveness and accuracy for lane detection. Methods such as SCNN [3] and RESA [4] regard lane detection as a segmentation task, which results in high computational complexity and poor real-time performance due to predicting each pixel individually. BézierLaneNet [5] took a different route by employing the Bézier curve to model lane markings, leading to the proposal of the feature flip fusion, which is an innovation that partly inspired our work. On another front, UFLD [6,7] aims at enhancing the speed of inference and proposes a row-wise classification method. LaneATT [8] proposed an anchor-based detection method and achieved good results in accuracy.

Although these methods have achieved favorable results, we identify several pressing issues that remain unaddressed. Firstly, the symmetrical features of images have not been fully utilized. Secondly, useful position information for locating lane lines still needs to be explored. Thirdly, obtaining contextual information from the surrounding environment can assist in lane detection. At the same time, in constantly changing road conditions and scenes, it is necessary to improve the accuracy and robustness of the detection model. Our work aims to tackle the outstanding challenges and push the boundaries of lane detection technology further.

We posit that lane lines inherently possess geometric properties, particularly manifesting in symmetrical attributes. Lane markings typically appear not as isolated entities but in dual arrangements. Specifically, upon the observation of a right-side lane marking, it is reasonable to infer the presence of a corresponding left-side marking, an idea that resonates with BézierLaneNet [5]. However, ref. [5] did not delve extensively into this overarching structural characteristic. In our research, we hypothesize that post-pixel alignment flipped features have a profound correlation with the original input features. To capitalize on this insight, we introduce the Flipped Feature Extraction (FFE) module. Within this module, we employ dilated convolutions at varying rates to generate multi-scale receptive fields, thereby capturing rich contextual and advanced semantic information. This strategic design aids us in enhancing the detection of target objects with greater precision and reliability.

**Figure 1:**
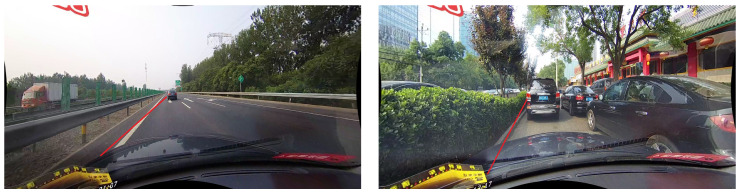
Inaccurate lane localization.

For lane line detection tasks, the process not only involves the identification of lane lines but also requires a precise determination of their positions, rendering position information critical to the accuracy of lane detection. As shown in Figure 1, it detected the presence of the lanes, but it did not accurately predict their locations. Despite its significant significance, the optimization and role of location information have not been widely discussed in the literature. In this work, we delve into the enhancement and utility of position information for lane detection and we propose the Hierarchical Position Information Extraction (HPIE) module, which encodes positional information in both the horizontal and vertical directions and strengthens the discriminative power of location cues. Our HPIE module effectively integrates positional information across the feature, thereby improving the precision of both detection and localization, ultimately contributing to a more accurate portrayal of lane line positions within the given scene.

We also propose a novel Deformable Context Extraction (DCE) module that is designed to meticulously extract fine-grained information from the surrounding environment. Its capability aids significantly in detecting lane line objects by focusing on salient and discriminative features. The DCE module not only outputs relevant response features but also effectively filters out noise interference, thereby enhancing the robustness and precision of the detection process. It contributes to an overall improvement in the system’s lane line detection capabilities, especially under complex and challenging scenarios where discerning between actual lane lines and potential distractions is crucial.

In this paper, we integrate the aforementioned modules into our proposed network architecture: FF-HPINet. The main contributions of this paper can be summarized as follows:

- We propose the Flipped Feature Extraction (FFE) module, which models the symmetric properties of lane lines and utilizes multi-scale receptive fields to collect contextual information, establishing effective interaction between flipped features and original features, enhancing the detection of target objects.
- We propose the Hierarchical Position Information Extraction (HPIE) module, which effectively aggregates positional information within feature maps, enhancing localization precision.
- We propose the Deformable Context Extraction (DCE) module, which meticulously extracts subtle and fine-grained information from the complex surrounding environment. It is adept at identifying and outputting relevant response features that are crucial for accurate lane line detection, boosting the overall performance and reliability of our proposed lane detection model.
- Our proposed FF-HPINet has demonstrated excellent performance on TuSimple and CULane datasets, achieving remarkable results in the field of lane detection.

## 2. Related Work

### 2.1. Lane Detection

The frameworks for lane detection in deep learning can be categorized into three main types: segmentation-based, anchor-based, and parameter-based approaches.

The segmentation-based approach involves treating the lane detection task as a semantic segmentation problem, where each pixel is classified to determine whether it belongs to a lane line or not. This approach includes models such as SCNN [3], RESA [4], LaneNet [9], and [10,11,12,13,14,15]. The CurveLanes-NAS [16] utilize neural architecture search (NAS) to search for a better network while requiring high GPU hours. These methods rely on pixel-by-pixel prediction of the entire image, resulting in high computational complexity. They require a significant amount of GPU time and might not deliver real-time performance.

In the domain of anchor-based approach, Line-CNN [17], LaneATT [8], and UFLD [6,7] are notable examples, with the Line-CNN being the pioneer of this approach. They use predefined anchors to identify potential locations of lane markings. Instead of segmenting every pixel, this method focuses on classifying and regressing anchor boxes that are likely to contain lane segments, which typically reduces computation compared to segmentation methods and can improve real-time performance since it narrows down the search space for detecting lanes. However, these methods do not perform well enough in challenging scenarios and cannot effectively address detection challenges in complex scenarios.

The work [18] pioneers the parameter-based approach, wherein polynomial curves are leveraged to model lane markings. PolyLaneNet [19] takes a direct route by applying polynomial regression for the prediction of these parameters. LSTR [20] adopts transformers [21] for predicting polynomials. Meanwhile, BézierLaneNet [5] resorts to the Bézier curve in its regression method for lane line delineation. Despite the inherent advantage of parameter-based methods in delivering faster processing speeds, their overall performance often does not excel compared to alternative methodologies.

### 2.2. Context Information

Lane markings are typically found in specific environments, and the surrounding contextual information has been proven instrumental in enhancing detection accuracy. The work [22] suggests that effective contextual information can be employed to aid in the detection of the target object. Moreover, ref. [23] demonstrates a notable improvement in detection accuracy through the collection of contextual information. Further to this line of inquiry, ContextNet [24] introduces a ContextNet module designed to capture and harness the contextual information encircling the proposal region, thereby contributing to enhanced detection performance. Bell et al. propose leveraging recurrent neural networks (RNNS) to gather and integrate both internal and external information from within the proposal area in the Inside–Outside Net (ION) [25]. Chen et al. propose a context-aware refinement algorithm that significantly enhances the precision of object proposals across various regions through the meticulous extraction and utilization of rich contextual information [26]. Furthermore, Chen et al. propose a knowledge graphs framework for exploiting relational and contextual information to infer occluded objects [27]. In VSSA-NET, Yuan et al. make modifications to the Long Short Term Memory (LSTM) network to encode the contextual features [28]. Recently, vision Transformer [29] has gained significant popularity in the field of computer vision. It is capable of gathering global information and establishing long-range dependencies. Consequently, numerous studies have been proposed. On this basis, many works [30,31,32,33] have been proposed, promoting its application in the field of vision. The design of these methods is relatively complex, we adopt a relatively simple but effective design.

## 3. Method

**Figure 2:**
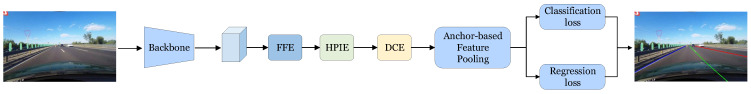
The overview of our FF-HPINet for lane detection pipeline.

In this section, we will introduce the lane detection network: FF-HPINet, as shown in Figure 2. Subsequently, we will introduce the details of the modules designed in our proposed lane detection method.

### 3.1. Network Overview

The FF-HPINet shown in Figure 2 consists of a backbone, a Flipped Feature Extraction (FFE) module, a Hierarchical Position Information Extraction (HPIE) module, a Deformable Context Extraction (DCE) module, and following an anchor-based feature pooling, the classification loss and the regression loss.

### 3.2. Flipped Feature Extraction

Given that the input image is captured by a camera mounted at the front of the car, our primary focus lies in exploiting the symmetry property of the lane lines. Specifically, the existence of a right lane line often indicates the presence of a corresponding left lane line. To capture this symmetry, we utilize the flip feature technique. The Feature Flip Fusion proposed in [5] has demonstrated promising results, but we recognize that the fusion may be considered rudimentary and lacks a thorough exploration of the relationship between the flipped and original features.

**Figure 3:**
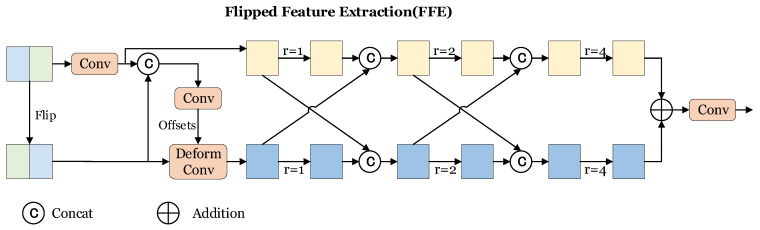
Architecture of Flipped Feature Extraction (FFE).

To address this limitation and enhance the extraction of semantic context from larger receptive fields, inspired by [5,34], we introduce our Flipped Feature Extraction (FFE), shown in Figure 3, which aims to better leverage the symmetry feature of lane lines while capturing rich contextual information from different receptive fields. By doing so, we aim to improve the accuracy and reliability of lane line detection in complex road environments.

In the images captured by the on-board front camera, the feature alignment process becomes necessary due to the incomplete left-right symmetry. To achieve this, we start by horizontally flipping the original feature $X\in R^{C\times H\times W}$ and concatenate it with the original feature for the network to learn the offsets required for feature alignment, it can be formulated as

(1) $$X_{flip}=X.\mathrm{flip}\left(-1\right),$$

(2) $$X_{0}=\mathrm{Conv}\left(X\right),$$

(3) $$offsets=\mathrm{Conv}(\mathrm{Cat}\left(X_{0},X_{flip}\right)).$$

Then, deformable convolution [35] is applied on the flipped feature, resulting in the acquisition of a modulated flipped feature:

(4) $$X_{flip}^{\prime }=\mathrm{DeformableConv}\left(X_{flip},offsets\right).$$

To extract rich contextual information and the several feature maps in different receptive fields from the two features, we employ multi-path dilated convolutional layers [23] with varying dilation rates [34]. In our approach, we employ three different dilation rates: 1, 2, and 4. To effectively model contextual dependencies between the original and flipped features, we incorporate cross connections between them. This involves splicing each feature map in the original branch with a pre-flipped feature map from the flipped branch before passing them through a dilated convolution layer. For example, before the feature fed into the second convolutional layer, we concatenate the feature:

(5) $$X_{1}=\mathrm{Cat}\left({\mathrm{Conv}}_{r=1}\left(X_{0}\right),X_{flip}^{\prime }\right),$$

(6) $$X_{2}=\mathrm{Cat}\left(X_{0},{\mathrm{Conv}}_{r=1}\left(X_{flip}^{\prime }\right)\right).$$

This process is repeated before the convolutional layers. By creating cross connections between the original and flipped features, we allow for continuous interactions and splicing, facilitating the propagation of features and maximizing the extraction of rich semantic attributes. This approach also helps establish contextual dependencies between the two branches. Concurrently, the use of dilated convolution layers with different dilation rates facilitates the fusion of features from multiple receptive fields.

Finally, to maintain the original feature map’s dimension and improve discrimination between foreground and background information, we sum the high-level semantic feature maps from both branches. The feature map is then fed into a 1×1 convolutional layer,

(7) $$X_{out}=\mathrm{Conv}\left(X_{1out}+X_{2out}\right),$$

which ensures the network can effectively discern between different regions of interest in the feature map while preserving the overall structure.

### 3.3. Hierarchical Position Information Extraction

**Figure 4:**
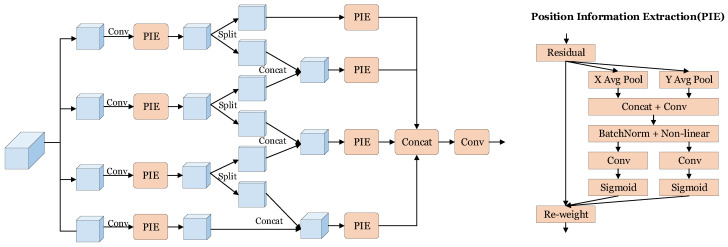
Architecture of Hierarchical Position Information Extraction (HPIE).

The image feature extracted from FFE contains rich contextual information, which helps to distinguish foreground from background; however, it cannot help us to improve the accuracy of localization of the lanes. To solve this problem, inspired by [36,37,38], we propose Hierarchical Position Information Extraction (HPIE), as shown in Figure 4, which aims to collect accurate position information. We will commence by elucidating Position Information Extraction (PIE), an integral precursor and component of our proposed HPIE, followed by a comprehensive overview of our proposed HPIE.

Positional information is pivotal for accurate localization and essential in effectively capturing the objects of interest. Consequently, we incorporate the design in [36] to build our PIE to augment the discriminative power of location information and thereby achieve more precise object localization.

We apply average pooling along the horizontal and vertical directions to obtain the global position information encoding,

(8) $$F_{h}={\mathrm{AvgPool}}_{x}\left(F\right),$$

(9) $$F_{W}={\mathrm{AvgPool}}_{y}\left(F\right).$$

Next, we concatenate the feature maps generated by the encodings from (8) and (9). The concatenated feature maps are then processed through convolutional layers, followed by a non-linear activation function, resulting in the feature map $F^{\prime }$:

(10) $$F^{\prime }=\mathrm{NonLinear}\left(\mathrm{Conv}\left(\mathrm{Cat}\left(F_{h},F_{w}\right)\right)\right).$$

The feature map $F^{\prime }$ encodes and merges the rich position information globally. Subsequently, we split the feature map $F^{\prime }$, both are then activated independently through convolutional layers and the sigmoid function, which enables us to obtain $F_{h}^{\prime }$ and $F_{w}^{\prime }$ and preserve accurate positional information along horizontal and vertical directions, respectively. By utilizing $F_{h}^{\prime }$ and $F_{w}^{\prime }$ as weights, which are multiplied with the original input feature,

(11) $$F^{\prime \prime }=F\times F_{h}^{\prime }\times F_{w}^{\prime },$$

we generate a feature map that effectively embeds the global positional information within its representation.

In order to improve the effectiveness of location information extraction, we intend to apply the PIE module multiple times. We ingeniously integrated PIE into HPIE while promoting cross channel integration of information, deepening the architecture of the model, while maintaining its accuracy in extracting location information.

Specifically, we split the feature map equally along the channel dimension into *m* groups, denoted as $f_{i}\in R^{C/m\times H\times W}$, where i=1,2,…,m. For each group, the feature map is fed into a convolutional layer followed by a PIE module. Subsequently, we split it into two parts $f_{i,1}$ and $f_{i,2}$, and concatenate them with the features from the preceding and succeeding branches respectively and the feature passes through the PIE module again:

(12) $$f_{i}^{\prime }=\left\{\begin{array}{cc} \mathrm{PIE}(f_{i,1})\; & i=1\\ \mathrm{PIE}(\mathrm{Cat}\left(f_{i-1,2},f_{i,1}\right))\; & 1<i<m\\ \mathrm{PIE}(\mathrm{Cat}\left(f_{i-1,2},f_{i}\right))\; & i=m \end{array}\right.$$

Through this approach, the current group can effectively utilize the position information obtained from the other group, enabling rich reuse of location information. This facilitates sufficient communication between groups, ultimately leading to improved accuracy of the location information. In addition, it also facilitates cross channel information fusion and increases model depth. By allowing groups to share and exchange relevant location information, the effectiveness of location information has been improved.

### 3.4. Deformable Context Extraction

**Figure 5:**
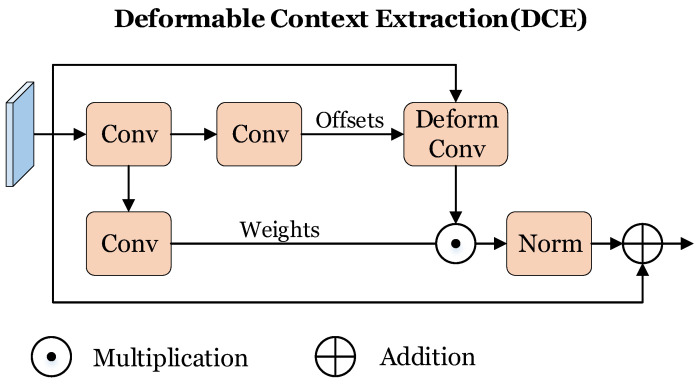
Architecture of Deformable Context Extraction (DCE).

In this section, we introduce the Deformable Context Extraction (DCE) module based on deformable convolution [35], as shown in Figure 5, which is designed to effectively extract foreground details and contextual information from the surrounding environment, thereby achieving local pixel alignment and significantly enhancing the precision of lane detection.

Given an input feature $Y\in R^{C\times H\times W}$, the intermediate feature map $Y_{0}$ is first obtained through convolutional layers

(13) $$Y_{0}=\mathrm{Conv}\left(Y\right).$$

Two independent convolutional layers are employed to compute the offsets *O* and the weights *W*, and can be formulated as:

(14) $$O=\mathrm{Conv}(Y_{0}),$$

(15) $$W=\mathrm{Conv}(Y_{0}).$$

The offsets *O* refers to the deformable convolution sampling offsets, the weights *W* represent the intensity response of each pixel within the feature map. The original input feature and the computed offsets are jointly fed into the deformable convolution layer to derive a feature map encapsulating the rich surrounding information. By multiplying this feature map with the weights, that is

(16) $$Y^{\prime }=W\cdot \mathrm{DeformableConv}\left(Y,O\right),$$

it selectively strengthens the relevant response features while suppressing non-relevant ones, thereby improving the discriminative power of the feature responses. Finally, a residual term is added to the feature map to address the issue of vanishing gradients,

(17) $$Y_{out}=Y+Norm\left(Y^{\prime }\right),$$

ensuring effective backpropagation during the learning process and further boosting the overall performance of our model in detecting lane lines accurately.

## 4. Experiments

### 4.1. Datasets

To evaluate the performance of our proposed method, we conducted experiments on two widely used lane detection benchmark datasets: TuSimple [39] and CULane [3]. The TuSimple dataset is taken under good weather conditions with stable lighting conditions, which is relatively easy. It comprises high-quality images captured by a car driving on California highways. It includes 3626 images for training and 2782 images for testing, all of which have dimensions of 1280×720 pixels. The CULane dataset is a large-scale dataset; it is collected from urban and highway scenes, covering nine different challenging scenarios, i.e., normal, crowded, dazzle, shadow, no line, arrow, curve, cross, and night conditions. It consists of 88,880 images for training and 34,680 images for testing, all of the image are 1640×5920 pixels.

### 4.2. Evaluation Metrics

The evaluation metrics for the TuSimple dataset include accuracy, false positive rate (FP), and false negative rate (FN); the accuracy is calculated by:

(18) $$accuracy=\frac{\sum_{clip}C_{clip}}{\sum_{clip}S_{clip}},$$

where $C_{clip}$ is the number of points predicted correctly, and $S_{clip}$ is the number of ground truth points in the clip. A predicted point within 20 pixels of the ground truth points is considered correct, and the predicted lane is considered as a true positive if the accuracy is greater than 85%.

For CULane, the lanes are considered to be 30 pixels wide. If the intersection-over-union (IoU) between predictions and ground truth is larger than 0.5, the predicted lanes are considered true positives. We also use the rate of false negative (FN) and false positive (FP) to evaluate our method.

Another evaluation metric we use is the *F*1 score, it is formulated as:

(19) $$F1=\frac{2\times Precision\times Recall}{Precision+Recall},$$

where $Precision=\frac{TP}{TP+FP}$, $Recall=\frac{TP}{TP+FN}$.

### 4.3. Implementation Details

We use ResNet-18 and ResNet-34 [40] as the backbone networks to create different versions of our proposed FF-HPINet. The input resolution is 360 pixels in width and 640 pixels in height. During the training phase, we apply data augmentation techniques, which consist of random horizontal flips and random affine transformations. We set the number of training epochs differently: 15 epochs for CULane [3] and 100 epochs for TuSimple [39], with a batch size of eight images per iteration. The model optimization strategy employed is the Adam optimizer, initially set with a learning rate of 0.0003, and the learning rate decay follows the cosine annealing strategy. We experiment with three distinct dilated rates r=1,2,4 in the FFE module. The number of groups in the HPIE module is m=4.

### 4.4. Comparison Results

**Figure 6:**
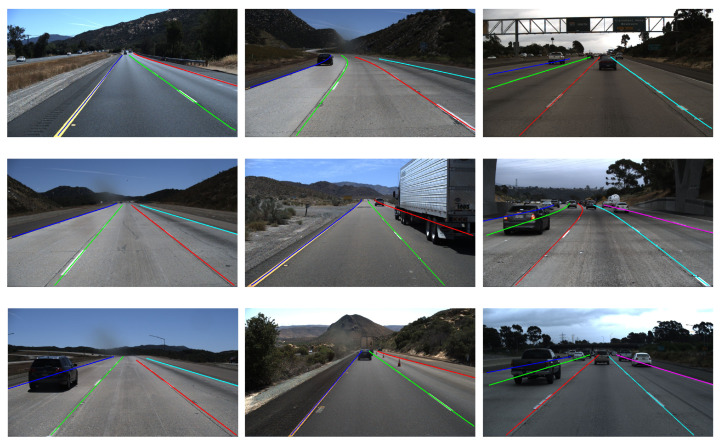
Visualization results of our FF-HPINet on TuSimple dataset.

We show our results on the TuSimple dataset in Table 1 and provide a visualization of the experimental results in Figure 6. As illustrated in Table 1, our proposed method obtained the highest F1 score. We observed that SCNN [3] achieved the best FN score of 1.80%, but its FP score was relatively poor at 6.17%. In comparison, our R18 version of FN achieved a commendable score of 2.81%, while our FP score remained good at 3.50%. Furthermore, our R34 version demonstrated a balanced performance with an FP score of 3.12% and an FN score of 2.88%, indicating a strong equilibrium between false positive and false negative. Due to the small scale of the dataset and the fact that the dataset was shot on a well-lit highway with simple scenes, other methods have already achieved impressive results with a small performance gap. However, our method still achieves a remarkable F1 score of up to 97.00%, which is significantly better than the other methods. The visualization results also demonstrate the robustness of our method. From the figure, it is evident that our predicted lane lines align precisely with the actual lane markings, demonstrating the efficacy of our proposed HPIE in assisting the network in precise lane line localization. In Figure 6, focusing on the following images, the image in the middle of the second column shows that the right lane is not fully visible due to vehicle obstruction; the image at the bottom of the first column shows the left lane line obstructed; the two images below the third column show vehicles blocking the lane lines on both sides of the view. As our proposed FFE can leverage the symmetric presence of lane lines, and our DCE can utilize contextual information from the surrounding environment, despite facing these visual obstacles, our method is still able to successfully and accurately identify the presence of lanes, demonstrating its strong robustness and accuracy.

**Figure 7:**
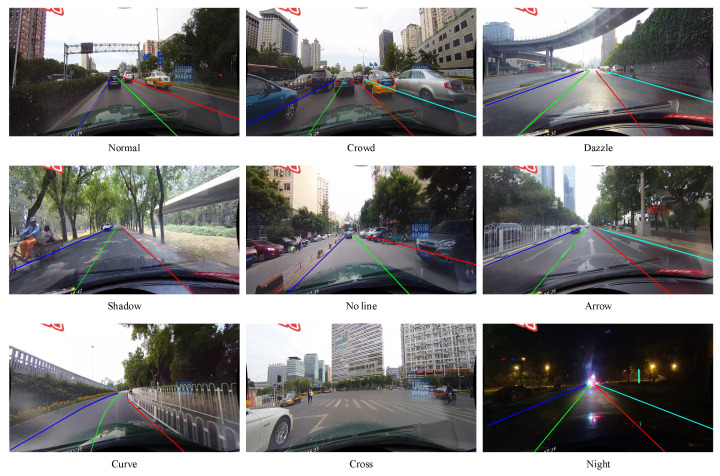
Visualization results of our FF-HPINet on CULane dataset.

The results of our FF-HPINet testing on the CULane dataset, as well as comparisons with other methods, are presented in Table 2. As shown in the table, our proposed method is significantly superior to other methods. The R34 version of our FF-HPINet achieved an impressive F1 score of 76.84%. Additionally, our approach has demonstrated commendable performance under crowd, no line, and night conditions. Our method attained an impressive score of 75.05% in crowd condition, 49.55% in no line condition, and 71.69% in night condition, outperforming other methods. The good performance signifies the method’s remarkable capability to effectively exploit the copious amounts of high-level semantic data embedded in the vicinity of lane lines under adverse conditions. As a result, our approach significantly enhances the precision and reliability of detecting and localizing these lane lines amidst a variety of challenging circumstances. The results of testing on the CULane dataset are shown in Figure 7. From Figure 7, our method effectively detects and locates lanes across all scenarios. Thanks to the HPIE module, our FF-HPINet predicted lane line trajectory overlaps almost perfectly with the white markings on the ground. Specifically, it is evident that our proposed method exhibits accurate lane line detection and localization even in challenging lighting conditions, such as dazzle light and night scenarios. Owing to the FFE module’s capacity to harness the inherent symmetry properties of the image, our method skillfully utilizes information from the lane line to accurately infer the position of the unseen lane line, detecting precise lane depiction. Furthermore, our method demonstrates resilience in environments where the lane lines are confounded by the presence of analogous semantic features on the road surface, such as those found in areas marked with directional arrows. It indicates that the DCE module enables our model to focus on areas related to lane markings while ignoring and suppressing interference information from arrows. This highlights the robustness and adaptability of our approach in handling complex and challenging real-world driving situations.

### 4.5. Ablation Study

To validate the effectiveness of our proposed modules, we carried out ablation studies utilizing the R18 version on the CULane dataset. The empirical outcomes of these experiments are systematically presented in Table 3, allowing for a comprehensive assessment of the contribution and effectiveness of our proposed modules.

#### 4.5.1. Effectiveness of FFE

As illustrated in Table 3, in Model 3, where the F1 score was originally 75.16%, the adoption of FFE led to an increase to 75.61% in Model 4—a significant uplift of 0.45%. Further, upon appending the FFE module to Model 5, we derived the FF-HPINet model, which achieved the superior F1 score of 78.85%, reflecting a net gain of 0.23%. Our FFE can leverage the characteristic of lanes existing in pairs, inferring the existence and position of the right lanes based on those on the left side of the vehicle. This is particularly useful in scenarios where there is a lack of visual representation of lane markings, thereby assisting the model in detecting them. The quantitative jump serves as empirical validation of the positive impact and effectiveness of the FFE module in enhancing the model’s capabilities.

#### 4.5.2. Effectiveness of HPIE

In Table 3, after incorporating the HPIE module into Model 3 and obtaining Model 5, there was a discernible improvement in performance as evidenced by the score leap from 75.16% to 75.62%, reflecting a net gain of 0.46%. Model 4, in contrast, without the inclusion of HPIE, achieved an F1 score of 75.61%. However, when equipped with the HPIE module, our FF-HPINet achieved a remarkable F1 score of 75.85%, showcasing its superior performance. Thanks to the enhanced positioning accuracy of lanes achieved by our HPIE, the model’s detection performance and robustness have been significantly improved.

#### 4.5.3. Effectiveness of DCE

As depicted in Table 3, our experiments demonstrate notable improvements in performance when incorporating the DCE module into the model. Particularly, with the integration of DCE, Model 4 achieved an improved F1 score of 75.61%, surpassing the Model 1 score of 75.36%. Compared with Model 2, Model 5 demonstrates a notable improvement by enhancing the F1 score from 75.44% to 75.62% upon the integration of DCE. These results highlight the efficacy of the proposed module in enhancing the performance of our models. It validates that the DCE enables the network to collect and utilize contextual information, focus more on lane line areas, and ignore interference information, thereby improving the detection accuracy of the model.

### 4.6. Analysis

**Setting of *r*.** In our experimentation, we varied the dilated rate across four different values, and the corresponding results are tabulated in Table 4. It is observed that when we configured the dilated rate r=1,2,4, the model delivered its optimal performance, attaining an F1 score of 75.85%. This peak performance is closely tied to the specific input image size chosen for our experiments. It is important to note that for alternative network architectures, a change in the predefined input size would likely necessitate adjustments to the optimal dilated rate setting to maintain or improve performance.

**Setting of *m*.** To investigate the effect of varying the group count in the HPIE module on the experimental outcomes, we carried out a series of tests, and the resultant data are compiled in Table 5. Upon setting the number of groups m=2, the model achieved an F1 score of 75.36%, concurrent with a peak FPS of 165. When doubling the number of groups m=4, the F1 score reached its zenith at 75.85%, albeit with a corresponding FPS reduction to 144. Nevertheless, we consider this FPS rate to be adequate for practical purposes. Upon further increment to eight groups, m=8, the F1 score dipped slightly to 75.66%. The slight decrease is due to excessive inter-group information fusion, which leads to the introduction of information redundancy and has a negative impact on overall performance.

### 4.7. Limitation and Discussion

The design of the Flipped Feature Extraction module is grounded in images captured by the front-facing camera. With a deep understanding of the underlying distribution patterns within the datasets, the model demonstrates outstanding adaptability and remarkable flexibility when dealing with images from such angles. However, its performance may be less effective when processing images captured by side-view or rear-view cameras, reflecting its inherent inductive bias. The adoption of image flipping implicitly assumes a certain degree of symmetry in the road environment and lane lines along the horizontal axis. This assumption holds true for the majority of road scenes across the datasets. However, in cases of significantly asymmetric road configurations or unique scenarios, such as temporary construction areas, the performance enhancement is marginally lower than in symmetrical scenarios.

In the dazzle scenario, when compared with the top-performing method BézierLaneNet, our model’s performance lags somewhat. While BézierLaneNet achieves an F1 score of 69.20%, our method garners a score of 66.80%—a difference of 2.4% lower. This disparity can be traced back to the core architectural decisions in our methodological approach. Our method is founded on detection principles and heavily depends on the textural information embedded within image features. Unfortunately, under severe lighting conditions, such textural details can become distorted or lost, leading to a decline in detection precision. On the contrary, BézierLaneNet takes a different route, predicting lane lines as continuous curves and representing them in a parameterized format. This strategy renders the model more resilient to variations in feature texture, enabling it to maintain a consistent level of performance under adverse lighting scenarios. In our ongoing research, we aim to tackle this specific issue head-on by refining our method to better cope with drastic lighting conditions and improve the robustness of our lane detection model.

Based on the data provided in Table 2, it is evident that not only our proposed method but also all comparison methods are difficult in the no line condition. Unlike situations where lane markings are partially or completely invisible due to lighting, occlusion, and other factors, the no line scene itself does not delineate lane markings, nor does it exhibit any discernible visual cues related to the lane. This is a significant obstacle for lane detection systems. In this case, the algorithm must utilize other contextual information, including inferring the existence and position of lane markings based on the vehicle’s heading, surrounding environment, and possible lane standard widths. In addition, the network also needs to estimate the range or length of lane markings, which requires advanced intelligent prediction and understanding of the driving environment. Finding solutions will be an interesting thing in future work.

Curved lane lines are a typical and frequent occurrence on many roads; however, the CULane dataset presents a certain degree of bias in that the majority of its lane lines are straight, with only a minority being curved. This inherent imbalance leads to the observation that all evaluated methods, including ours, demonstrate reduced performance in the curve scene. Despite this limitation, our method has nonetheless managed to deliver respectable results, achieving an F1 score of 68.06% in the curve scenario. Moving forward, we are committed to enhancing the model’s generalizability, aiming to optimize its performance on a wider variety of road geometries.

## 5. Conclusions

In this work, we introduce a lane detection network named FF-HPINet, which innovatively integrates unique architectural components to address the challenges in lane detection tasks. To begin with, acknowledging the intrinsic geometric symmetry of lane lines, we designed the Flipped Feature Extraction (FFE) module. This module exploits the symmetry property to forge strong connections between mirrored and original feature representations, thereby bolstering the network’s capacity to discern and localize lane regions of interest more effectively. Additionally, for precise localization of lane lines, we propose the Hierarchical Position Information Extraction (HPIE) module. The module ingeniously captures location information in both horizontal and vertical dimensions and partitions the features into multiple groups. This strategy not only enriches the reuse of positional information but also facilitates cross channel information fusion and increases model depth without compromising on the accuracy of location information. Furthermore, we design the Deformable Context Extraction (DCE) module, which excels in extracting foreground details and contextual information from the immediate environment. By achieving local pixel alignment, this module amplifies the intensity response of the targeted areas while suppressing irrelevant signals, thereby refining the network’s overall detection abilities and delivering enhanced performance in diverse and intricate scenarios. Experimental results on CULane and TuSimple datasets demonstrate the effectiveness of our FF-HPINet. In future research, we will continuously strive to improve the detection accuracy in complex scenes. Additionally, we will explore the application of self-supervised and weakly-supervised learning in the field of lane detection to enhance the model’s adaptive ability.

## Figures and Tables

**Table 1:** Comparison results on the TuSimple dataset.

Method	F1	Accuracy	FP	FN
SCNN [3]	95.97	96.53	6.17	**1.80**
LaneNet [9]	94.80	96.40	7.80	2.44
SAD-R18 [11]	93.79	96.02	7.86	4.51
SAD-R34 [11]	94.68	96.24	7.12	3.44
E2E-R18 [14]	96.40	96.04	3.11	4.09
E2E-R34 [14]	96.58	96.22	3.08	3.76
LSTR [20]	96.85	96.18	**2.91**	3.38
LaneATT-R18 [8]	96.71	95.57	3.56	3.01
LaneATT-R34 [8]	96.77	95.63	3.53	2.92
RESA-R18 [4]	96.61	96.70	3.95	2.83
RESA-R34 [4]	96.94	**96.82**	3.63	2.48
Eigenlanes [41]	96.40	95.62	3.20	3.99
UFLDv2-R18 [6]	96.16	95.65	3.06	4.61
UFLDv2-R34 [6]	96.22	95.56	3.18	4.37
BézierLaneNet-R18 [5]	95.05	95.41	5.30	4.60
BézierLaneNet-R34 [5]	95.50	96.65	5.10	3.90
FF-HPINet-R18	96.84	95.63	3.50	2.81
FF-HPINet-R34	**97.00**	95.67	3.12	2.88

R18 denotes ResNet-14, R34 denotes ResNet-34. The best results are in bold.

**Table 2:** Comparison results on the CULane dataset.

Method	Total	Normal	Crowd	Dazzle	Shadow	No Line	Arrow	Curve	Cross	Night
SCNN [3]	71.60	90.60	69.70	58.50	66.90	43.40	84.10	64.40	1990	66.10
SAD-R18 [11]	70.50	89.80	68.10	59.80	67.50	42.50	83.90	65.50	1995	64.20
SAD-R34 [11]	70.70	89.90	68.50	59.90	67.70	42.20	83.80	66.00	1960	64.60
CurveLane-L [16]	74.80	90.70	72.30	67.70	70.10	49.40	85.80	68.40	1746	68.90
E2E-R18 [14]	70.80	90.00	69.70	60.20	62.50	43.20	83.20	**70.30**	2296	63.30
E2E-R34 [14]	71.50	90.40	69.90	61.50	68.10	45.00	83.70	69.80	2077	63.20
LaneATT-R18 * [8]	74.81	90.91	72.66	65.28	70.59	47.89	85.16	62.72	1193	68.84
LaneATT-R34 * [8]	76.60	92.12	74.91	66.97	**77.75**	49.24	88.24	67.54	1313	70.55
RESA-R34 [4]	74.50	91.90	72.40	66.50	72.00	46.30	88.10	68.60	1896	69.80
LaneAF-ENet [42]	74.24	90.12	72.19	68.70	76.34	49.13	85.13	64.40	1934	68.67
UFLDv2-R18 [6]	75.00	91.80	73.30	65.30	75.10	47.60	87.90	68.50	2075	70.70
UFLDv2-R34 [6]	76.00	**92.50**	74.80	65.50	75.50	49.20	**88.80**	70.10	1910	70.80
BézierLaneNet-R18 [5]	73.67	90.22	71.55	62.49	70.91	45.30	84.09	58.98	996	68.70
BézierLaneNet-R34 [5]	75.57	91.59	73.20	**69.20**	76.74	48.05	87.16	62.45	**888**	69.90
FF-HPINet-R18	75.85	91.45	73.36	67.20	72.48	49.12	86.78	64.39	1002	70.35
FF-HPINet-R34	**76.84**	91.92	**75.05**	66.80	76.18	**49.55**	87.76	68.06	1061	**71.69**

* Results tested on our device using the weights provided by the author. The best results are in bold.

**Table 3:** Ablation study on the CULane dataset.

Model	FFE	HPIE	DCE	F1
1	✓			75.36
2		✓		75.44
3			✓	75.16
4	✓		✓	75.61
5		✓	✓	75.62
FF-HPINet	✓	✓	✓	**75.85**

**Table 4:** Setting of *r*.

r	F1	FPS
*r* = 1, 2, 4	**75.85**	144
*r* = 1, 2, 8	75.56	144
*r* = 1, 4, 8	75.63	144
*r* = 2, 4, 8	75.45	144

**Table 5:** Setting of *m*.

*m*	F1	FPS
*m* = 2	75.36	**165**
*m* = 4	**75.85**	144
*m* = 8	75.66	128

## Data Availability

The data presented in this study are available from the authors upon reasonable request.
